# Global trends and performances in diabetic retinopathy studies: A bibliometric analysis

**DOI:** 10.3389/fpubh.2023.1128008

**Published:** 2023-04-13

**Authors:** Huan Xiao, Jinfan Tang, Feng Zhang, Luping Liu, Jing Zhou, Meiqi Chen, Mengyue Li, Xiaoxiao Wu, Yingying Nie, Junguo Duan

**Affiliations:** ^1^School of Ophthalmology, Chengdu University of Traditional Chinese Medicine, Chengdu, China; ^2^School of Acupuncture-Moxibustion and Tuina, Chengdu University of Traditional Chinese Medicine, Chengdu, China; ^3^School of Acupuncture-Moxibustion and Tuina, Beijing University of Chinese Medicine, Beijing, China

**Keywords:** diabetic retinopathy, CiteSpace, VOSviewer, public health, bibliometric analysis

## Abstract

**Objective:**

The objective of this study is to conduct a comprehensive bibliometric analysis to identify and evaluate global trends in diabetic retinopathy (DR) research and visualize the focus and frontiers of this field.

**Methods:**

Diabetic retinopathy-related publications from the establishment of the Web of Science (WOS) through 1 November 2022 were retrieved for qualitative and quantitative analyses. This study analyzed annual publication counts, prolific countries, institutions, journals, and the top 10 most cited literature. The findings were presented through descriptive statistics. VOSviewer 1.6.17 was used to exhibit keywords with high frequency and national cooperation networks, while CiteSpace 5.5.R2 displayed the timeline and burst keywords for each term.

**Results:**

A total of 10,709 references were analyzed, and the number of publications continuously increased over the investigated period. America had the highest h-index and citation frequency, contributing to the most influence. China was the most prolific country, producing 3,168 articles. The University of London had the highest productivity. The top three productive journals were from America, and Investigative Ophthalmology Visual Science had the highest number of publications. The article from Gulshan et al. (2016; co-citation counts, 2,897) served as the representative and symbolic reference. The main research topics in this area were incidence, pathogenesis, treatment, and artificial intelligence (AI). Deep learning, models, biomarkers, and optical coherence tomography angiography (OCTA) of DR were frontier hotspots.

**Conclusion:**

Bibliometric analysis in this study provided valuable insights into global trends in DR research frontiers. Four key study directions and three research frontiers were extracted from the extensive DR-related literature. As the incidence of DR continues to increase, DR prevention and treatment have become a pressing public health concern and a significant area of research interest. In addition, the development of AI technologies and telemedicine has emerged as promising research frontiers for balancing the number of doctors and patients.

## 1. Introduction

Diabetic retinopathy (DR) is the primary cause of visual impairment worldwide and is a common complication of diabetes mellitus (1). According to a survey by the 74th World Health Organization (WHO), more than 420 million people suffered from diabetes in 2021, and this number is expected to increase to 578 million by 2030. The global incidence of diabetes continues to increase, leading to a corresponding increase in the number of people affected by DR (2). It is estimated that from 2020 to 2045, the number of DR patients globally will increase from 103.12 million to 160.5 million (3), with 44.82 million people experiencing vision problems. This has become a significant global public health and economic issue.

Diabetic retinopathy is the subject of extensive research, with vast literature making it difficult to identify the research emphasis and frontier. Therefore, a comprehensive retrospective analysis is crucial to understanding the development state, research hotspots, and future development trends of DR.

To achieve this, we used VOSviewer, a program that excels at constructing any type of text map, for literature-based cooperative network analysis, co-occurrence analysis, citation analysis, literature coupling analysis, and co-citation analysis (4). We also employed CiteSpace’s timeline view, which depicts the progress of scientific research and mutation detection to identify the frontiers of scientific study (5). By utilizing the literature metrology approach and two bibliometric tools, we analyzed DR-related literature from the WOS to provide pertinent information.

## 2. Method

### 2.1. Data sources and search strategy

We obtained all literature from the Web of Science (WOS), a leading global database of scholarly information founded in 1985. WOS includes authoritative and influential journals across a wide range of disciplines. We used the WOS core collection to ensure that high-quality academic journals were selected and searched the database from its establishment until 1 November 2022. To maximize precision while maintaining search sensitivity, we merged the title and abstract and excluded interference from the “early treatment of diabetic retinopathy study.” Our retrieval formula was as follows: {[TI = (“diabetic retinopathy”) OR AB = (“diabetic retinopathy”)] NOT [AB = (“early treatment of diabetic retinopathy study”) OR AB = (“early treatment diabetic retinopathy study”)]} OR {[TI = (diabet*) OR TI = (mellitu*)] AND [AB = (“early treatment of diabetic retinopathy study”) OR AB = (“early treatment diabetic retinopathy study”)]}. We limited the document types to articles, the language to English, and excluded retracted publications.

### 2.2. Data collection

We downloaded literature information in batches and recorded “Full Records and References Cited.” To integrate the downloaded bibliographic data into CiteSpace 5.5.R2 and VOSviewer 1.6.17, we named the downloaded file “download X.” We collected publication counts, citation frequency, and h-index of countries, institutions, and journals, along with primary information on the top 10 most cited articles from the WOS analysis results and citation reports. We also evaluated the impact factors (IFs) of important journals derived from the 2022 Journal Citation Reports (JCRs).

### 2.3. Data analysis

We imported the findings of the WOS analysis results and citation reports into Microsoft Excel to provide annual publication counts, major nations, institutions, and journals in charts. We condensed the most significant data from frequently referenced articles and presented the findings in a tabular format. We used VOSviewer to display collaboration networks of countries and high-frequency keywords, setting the node type successively as countries and all keywords, and different thresholds to display only the top 37 countries and top 60 keywords. National node weights were selected based on total link strength, and keywords node weights on documents. Both figures used network visualization as the image type. We visualized keyword timelines and burst keywords using CiteSpace, setting the period of analysis from 1996 to 2022 since the first relevant literature was published in 1996. The time-slicing was set to 1 year and the threshold to *N* = 50. The node type was set as the keyword. We used Pathfinder, pruning networks, and pruning the merged networks to simplify minor wiring, while the remaining parameters were set to default settings.

## 3. Results

### 3.1. Growth trends of publications

**Figure 1:**
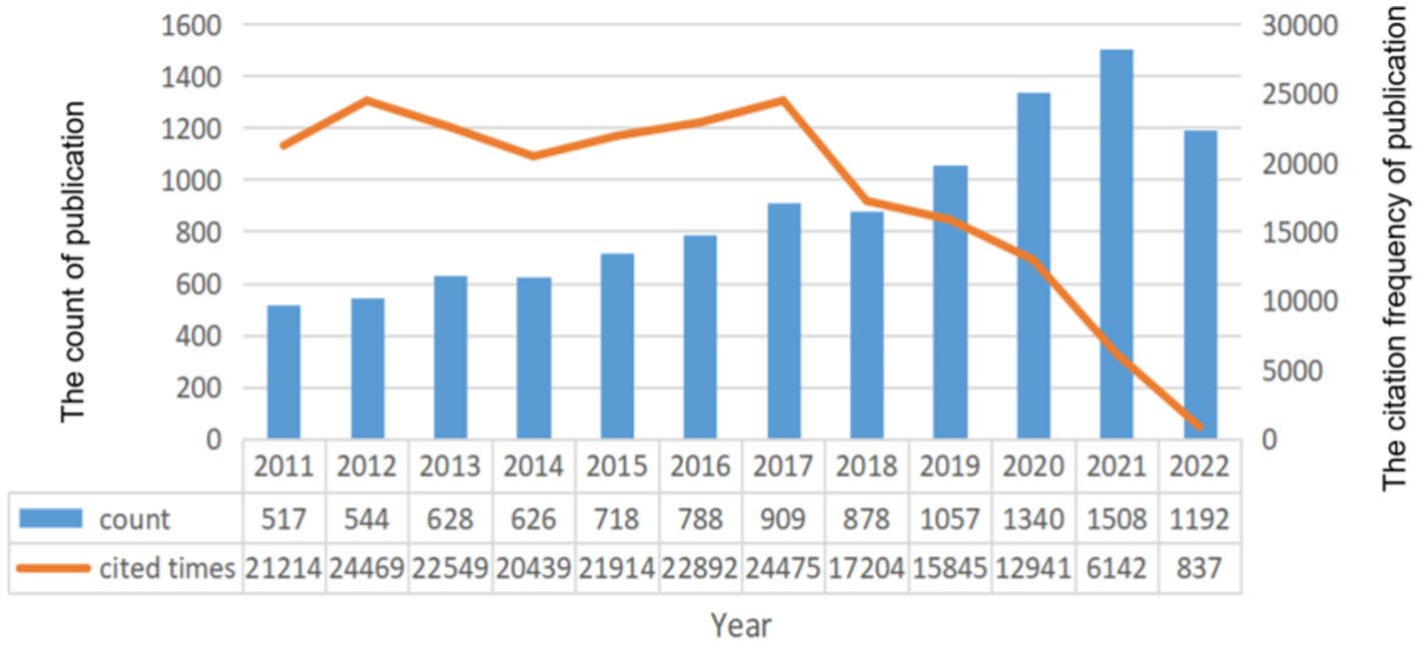
Distribution of annual years.

A total of 10,709 records met the search criteria. Jirousek MR et al. released the first record on the subject of DR in 1996. They proposed the idea of inhibiting overactive PKC isoenzyme to treat DR (6). However, at that time, few researchers paid attention to this field. From 1996 to 2010, there were only three scattered pieces of drug research literature (7, 8). In 2011, the number of publications on this topic skyrocketed. Since 2011, the number of publications regarding DR has typically increased, reaching a peak of 1,508 articles in 2021, and it may continue to increase this year (Figure 1). The top three citation frequency rankings were 2012, 2017, and 2016.

### 3.2. Distribution of countries

**Figure 2:**
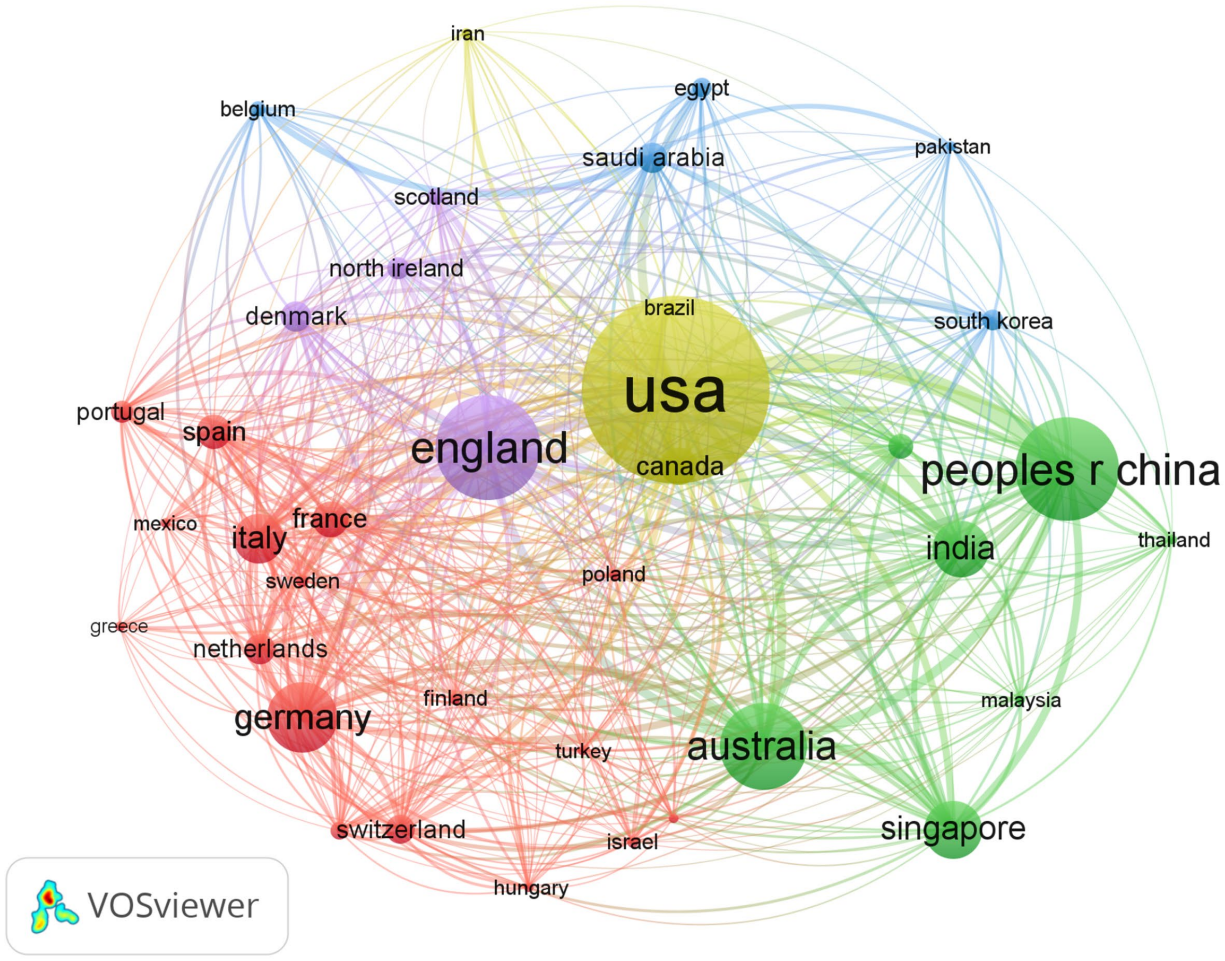
Bibliometric analysis of the countries’ cooperations on DR. The node represents a country, the bigger the node the total link strength is stronger. The lines represent cooperation, and the thickness of the lines represents the closeness of the cooperation.

According to the articles retrieved, relevant material was published in 127 countries. Table 1 displays the 10 nations with the most publications. The top three nations, China, America, and India, accounted for more than half of the total. Articles from America were cited 86,263 times, ranking first among all countries, followed by China and England. America and Germany had the greatest h-index and ACI, respectively. In addition, we conducted a study of country distribution and cooperation to assess the degree of international collaboration. Collaborations between nations were widespread, as seen in Figure 2. The cooperation map showed that America had the strongest total link strength (1,821) and the largest national cooperation network, followed by England and China.

**Table 1:** Top 10 countries by the volume of publication.

Country	C	P/%	*CF*	ACI	H-index	Total link strength
China	3,168	29.585	48,097	15.18	74	964
America	2,548	23.795	86,263	33.86	117	1821
India	822	7.677	18,281	22.24	51	595
England	688	6.425	21,128	30.71	56	1,056
Japan	602	5.622	14,840	24.65	48	231
Australia	516	4.819	18,050	34.98	58	870
South Korea	493	4.604	8,899	18.05	43	204
Italy	437	4.081	15,006	34.34	55	479
Germany	412	3.848	16,713	40.57	58	676
Spain	321	2.998	8,693	27.08	45	346

C, count; P, proportion; CF, citation frequency; ACI, average citation per item.

### 3.3. Distribution of institutions

The top 10 institutions in terms of the number of publications were from four distinct nations: England, America, China, and Singapore (Table 2). The University of London published the most articles, followed by Harvard University and Shanghai Jiao Tong University. According to the citation frequency analysis, the University of London in England had 13,035 citations, placing it at the top. The highest ACI and h-index were recorded by the Singapore National Eye Center and Harvard University, respectively.

**Table 2:** Top 10 institutions by the volume of publication.

Rank	Institution	Country	C	P/%	*CF*	ACI	H-index
1	University of London	England	332	3.100	13,035	39.26	47
2	Harvard University	America	265	2.475	12,251	46.23	50
3	Shanghai Jiao Tong University	China	262	2.447	4,327	16.52	33
4	University College London	England	251	2.344	8,101	32.27	42
5	National University of Singapore	Singapore	234	2.185	11,281	48.21	49
6	University of California System	America	227	2.120	8,573	37.77	40
7	Singapore National Eye Center	Singapore	223	2.082	11,128	49.90	48
8	Sun Yat Sen University	China	214	1.998	4,904	22.92	35
9	Harvard Medical School	America	208	1.942	9,541	45.87	43
10	Moorfields Eye Hospital Nhs Foundation Trust	England	196	1.830	5,511	28.12	37

### 3.4. Distribution of journals

Half of the top 10 most commonly circulated DR publications (Table 3) were from America, indicating the country’s significant impact. The British Journal of Ophthalmology had the highest IF (2022). Investigative Ophthalmology Visual Science published the most literature, which also had the highest citation frequency and ACI.

**Table 3:** Top 10 periodicals by the volume of publication.

Journal	C	P/%	*CF*	ACI	IF(2022)	Country
Investigative Ophthalmology Visual Science	461	4.305	16,587	35.87	3.458	America
PloS One	389	3.632	8,635	22.0	3.041	America
Retina the Journal of Retinal and Vitreous Diseases	245	2.288	6,446	26.31	3.394	America
Scientific Reports	240	2.241	3,557	14.82	4.130	England
Acta Ophthalmologica	178	1.662	2,800	15.73	2.702	America
British Journal of Ophthalmology	178	1.662	4,352	24.45	4.192	England
Graefes Archive for Clinical and Experimental Ophthalmology	167	1.559	2,455	14.70	2.811	Germany
Experimental Eye Research	156	1.457	2,311	14.81	3.161	America
International Journal of Ophthalmology	151	1.410	1,470	9.74	1.603	China
Eye	150	1.401	2,405	16.03	2.552	England

### 3.5. Top cited references

Table 4 provides a summary of the 10 most cited sources. These top 10 articles have been referenced over 11,000 times, with the first article being quoted 2,897 times and the 10th article being cited 541 times. The majority of these articles focused on deep learning and incidence.

**Table 4:** Top 10 literature with citation frequency.

Rank	Cited reference	C	The main contents of DR
1	Gulshan et al. (2016)	2,897	Develop algorithms to automatically detect DR and diabetic macular edema using deep learning (9).
2	Yau et al. (2012)	2,499	The global incidence and risk factors of DR, proliferative diabetic retinopathy (PDR), VTDR, diabetic macular edema (10).
3	Tabák et al. (2012)	1,397	Changes of retinal vascular, such as decreased arteriolar-to-venular ratio and increased retinal arteriolar or venular caliber, are present in prediabetes (11).
4	Bourne et al. (2013)	965	The worldwide rate of DR blindness was 1.3% in 1990 and 1.9% in 2010, when DR was not a major cause of blindness (12).
5	Ting et al. (2017)	877	A deep learning system using DR retinal images from multiethnic populations is developed and validated,which has high sensitivity and specificity (13).
6	Tang et al. (2011)	725	Review of the relationship between inflammatory mediators and DR (14).
7	Marin et al. (2011)	606	A new digital monitoring method for vascular detection in retinal images can be used for automatic screening of early DR (15).
8	Decenciere et al. (2014)	549	Messidor, a public database with a large number of DR fundus images, has been used more frequently (16).
9	Brown et al. (2013)	546	A 36-month injection of ranibizumab to diabetic macular edema patients resulted in a sustained improvement in retinal anatomy and best corrected visual acuity (BCVA) (17).
10	Gargeya et al. (2017)	541	A automatically diagnostic technology for DR was developed and evaluated,which achieved 94% sensitivity and 98% specificity (18).

### 3.6. Main keywords

**Figure 3:**
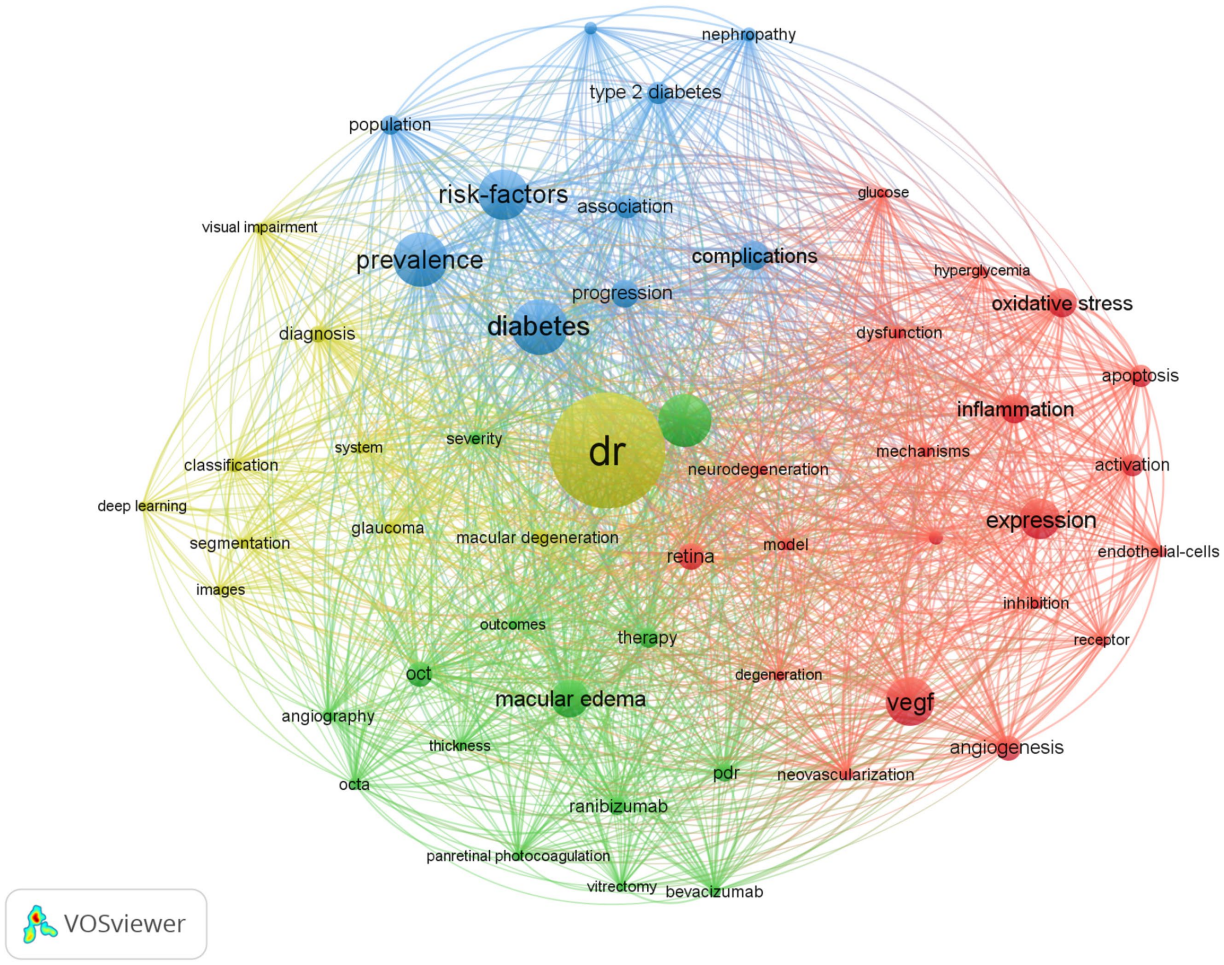
Bibliometric analysis of the keywords on DR. The node size represents the frequency of keyword occurrence, and the line represents the co-occurrence relationship. The same color is the keyword of the same cluster category.

Keywords can refine literature and help identify the field’s hotspots based on their frequency. The VOSviewer node was set to all keywords, and synonyms such as diabetes and mellitus, vegf and vascular endothelial growth factor were combined. Eventually, we identified the top 20 keywords (Table 5), and the top 60 are shown in Figure 3. Apart from “DR,” “diabetes,” and “retinopathy,” “prevalence” and “risk factors” were the most frequently occurring. Figure 3 shows four colors to illustrate the four primary research directions of DR: green for therapy clustering, red for pathogenesis clustering, yellow for AI clustering, and blue for epidemiology clustering.

**Table 5:** Top 20 keywords with frequency detected by VOSviewer.

Rank	C	Keyword	Rank	C	Keyword
1	4,755	Diabetic retinopathy	11	745	Inflammation
2	1827	Diabetes	12	712	Progression
3	1746	Prevalence	13	680	Retina
4	1,690	Retinopathy	14	608	Oct
5	1,586	Risk factors	15	607	Angiogenesis
6	1,494	Vegf	16	564	Association
7	1,161	Expression	17	548	Type 2 diabetes
8	1,044	Macular edema	18	529	Activation
9	781	Oxidative stress	19	521	Apoptosis
10	778	Complications	20	445	Population

### 3.7. The evolutionary path of keywords

**Figure 4:**
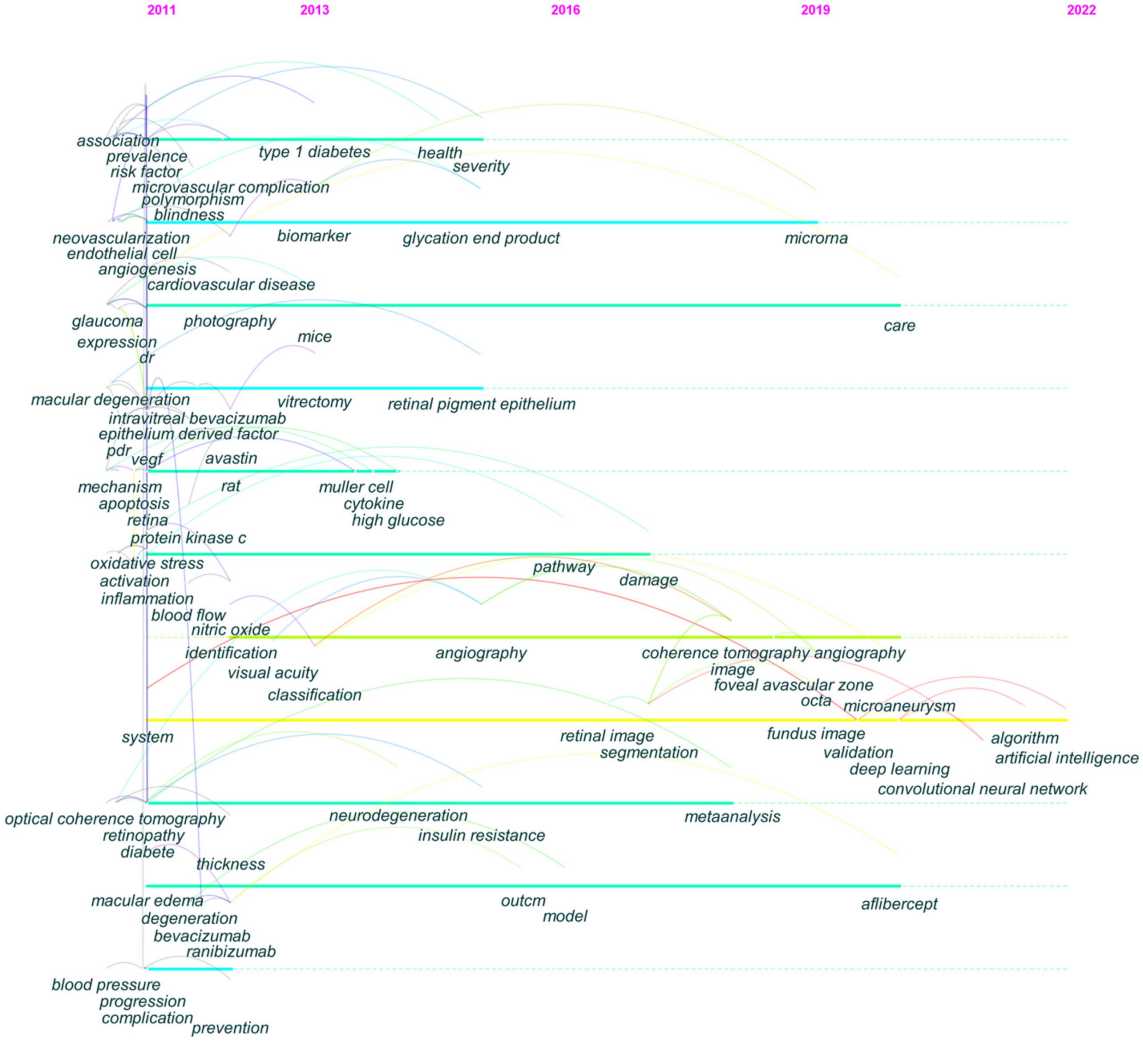
The evolutionary path of keywords detected by CiteSpace. The corresponding year of each node keyword was the year of high-frequency occurrence of the keyword.

Figure 4 depicts the distribution of keywords over time. By following each keyword transformation, the migration route of study emphasis can be seen intuitively. Prior to 2013, DR research focused on macular edema, risk factors, and neovascularization, among others. Between 2013 and 2016, biomarkers and neurodegeneration received significant attention. From 2016 to 2019, the focus shifted to OCTA, meta-analysis, and the foveal avascular zone (FAZ). During 2019–2022, AI and deep learning emerged as new research priorities.

### 3.8. Research frontier analysis

**Figure 5:**
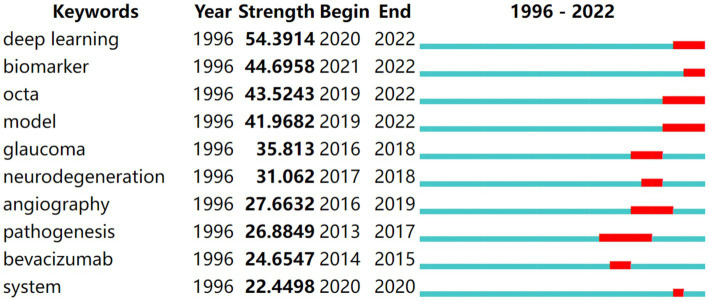
Top 10 keywords with the strongest citation bursts in DR research. The blue lines represent the base timeline, while the red segments represent the duration of the keyword burst, and the two endpoints correspond to the beginning and end times of the burst.

Keyword bursts, which illustrate terms that abruptly and often emerge within a certain period, can be used to forecast hotspot changes. Figure 5 presents the top 10 keywords with the strongest bursts. Deep learning had the strongest strength and was a crucial research focus from 2020 to 2022. Pathogenesis had the longest burst duration and was a research hotspot from 2013 to 2017. Deep learning, biomarkers, OCTA, and models were in the burst phase, indicating that they are the present research hotspots and may become the focus in the next few years.

## 4. Discussion

Bibliometric analysis is a prominent method to identify active research hotspots and future trends. It can visualize the relationships between the literature as a scientific knowledge map and is widely recognized as a valuable tool for mining useful information from the complex network structure of literature data (5). In this study, we conducted a bibliometric analysis of DR-related literature from the WOS, identifying publication trends and global contributions, and determining four clusters and keyword bursts within the visual network.

### 4.1. Publication trend in DR research

The trend changes in the number of publications reflect the updating of knowledge about a subject. Before 2011, DR had not received sufficient attention, as fewer articles were published. However, with the increasing number of diabetics in the world, the incidence of DR is also significantly increasing. The increasing incidence of DR and the advancement and promotion of fundus examination equipment have both contributed to the expansion of DR research. Since 2011, the research on DR has been in a rapid development stage, as reflected by the number of published articles. Based on the posting trend, it is predicted that the number of publications will reach a new peak this year. In addition, the citation frequency in 2017 is the highest, indicating that the research results have gained widespread attention.

### 4.2. International cooperation

According to the h-index and citation frequency, America is the leading country in DR research with the most impact, accounting for 23.795% of the total publications. China ranks second, with the largest number of publications but lesser influence. As research deepens, global cooperation is becoming closer. Based on the connections between the various nodes, America places great importance on communication and collaboration in academia, which explains why America has both high productivity and high-quality research. The top three prolific journals were all from America, with Investigative Ophthalmology and Visual Science (IOVS) being the most productive and cited journal in DR research. Among the top 10 prolific institutions, six were located in Europe and America, which is consistent with the increasing prevalence of DR in developed countries.

### 4.3. Research directions of DR

The keyword analysis results from VOSviewer revealed four clusters in the DR field. Based on the findings of the bibliometric analysis, we discuss the following four main research fields of DR research:

#### 4.3.1. Prevalence and risk factors of DR

In recent years, the prevalence of diabetes has been steadily increasing, leading to an increase in the incidence of DR, a serious worldwide public health concern. DR is the leading cause of blindness among individuals of working age (20–74 years old) in many countries (19). According to the International Diabetes Federation (IDF), in 2021, 537 million people aged 20–79 years have diabetes, accounting for 10.5% of the global population in this age group (20). Studies show that 22.27% of diabetes patients suffer from DR, and the prevalence of DR in diabetic individuals over a 10-year period may reach 60% (3). The prevalence of any DR is higher in individuals with type 1 diabetes (77.3%) than in those with type 2 diabetes (25.2%) (21). Available data suggest that 93 million people had DR in 2010 and 28 million were at risk of visual impairment (10). However, recent studies show that 860,000 people over the age of 50 worldwide will be blinded by DR in 2020 (22). By 2045, there will be 160.5 million DR patients, and 44.82 million patients may be at risk of visual impairment (3).

Multiple factors influence the occurrence and progression of DR. The incidence rates of DR vary depending on the region and the type or duration of diabetes (23). In addition, reviews suggest that the most significant risk factors for the initiation and progression of DR include smoking, a high body mass index, insufficient glycemic control, hypertension, a long history of diabetes, dyslipidemia, and microalbuminuria (24, 25). Arterial stiffness has also been proposed as a risk factor related to DR in recent years (26). Although the exact role of these factors in the pathogenesis of DR is not well-defined, they are important in guiding DR screening and the development of public healthcare strategies.

#### 4.3.2. Pathogenesis of DR

Advances in research have improved our understanding of the pathophysiological processes that lead to the development of DR. Hyperglycemia, along with other pathological risk factors such as hypertension, sets off a cascade of metabolic pathways that eventually cause microvascular damage and retinal malfunction.

The modification of the retinal microvasculature is the primary pathogenic feature of DR. The blood–retinal barrier, formed by tight junctions between endothelial and pericyte cells, is crucial for intravascular homeostasis (27). In a high glucose environment, abnormal expression of *circRNA-CZNF532* (28), *circEhmt1* (29), and *miRNA-138-5p* (30) induces pericyte degeneration and vascular dysfunction. The aberrant expression of *miRNA-34a* (31), *miRNA-126* (32), and *miRNA-221* (33) results in the upregulation of pro-inflammatory molecules, leading to the accumulation of leukocytes around the retinal capillary wall and apoptosis of vascular endothelial cells. Hyperglycemia also causes oxidative stress, which further leads to apoptosis, inflammation, and structural and functional changes in the retina (34, 35). Microangiopathy may cause damage to pericytes and endothelial cells, resulting in ischemic changes in the retina. The ischemic condition triggers compensatory mechanisms, resulting in the proliferation of vascular endothelial cells and the promotion of angiogenesis. The activation of *miRNA-21* (36) and the downregulation of *miRNA-200b* signify the eventual development of proliferative diabetic retinopathy (PDR) through neovascularization (37).

Furthermore, DR pathogenesis involves retinal neurodegeneration. Studies have shown that diabetic individuals have abnormal retinal neurons and glial cells, and retinal neurodegeneration occurs before retinal microvascular damage (38, 39). Retinal ganglion cells, photoreceptors, peakless cells, and bipolar cells all undergo changes during retinal neurodegeneration (40). Microvascular dysfunction and retinal neurodegeneration occur simultaneously (41). Along with research into the retinal vascular and neurological causes of DR, targeted drugs have been developed (42, 43).

#### 4.3.3. Treatment of DR

The effective regulation of glucose levels and other perilous aspects is paramount in forestalling the onset of DR during the diabetic phase. Sodium-dependent glucose transporter 2 inhibitors (SGLT2i) effectively regulate blood glucose levels and inhibit the manifestation of SGLT2 within the retina, thus mitigating the probability of DR (44). In addition, traditional Chinese medicine (TCM) monomers such as Lycium barbarum polysaccharide (45), Astragalus polysaccharide (46), curcumin (47, 48), and Crocin (49) can serve as natural extracts that can help protect the retina against apoptosis, inflammation, and oxidative stress. The combination of TCM monomers with nanotechnology can also address the issue of inadequate bioavailability, making them a promising solution for the early prevention and treatment of DR (50).

For individuals with a confirmed diagnosis of proliferative diabetic retinopathy (PDR), the main therapeutic options are retinal laser photocoagulation and intravitreal anti-vascular endothelial growth factor (VEGF) injection. Laser photocoagulation reduces VEGF production and the risk of vision loss by destroying the ischemic retinal area. Anti-VEGF treatment, on the other hand, improves retinal edema and patients’ vision by suppressing vascular permeability. Common anti-VEGF drugs include conbercept, aflibercept, ranibizumab, and bevacizumab. Intravitreal anti-VEGF injection has been found to significantly reduce peripheral vision impairment and decrease the incidence of macular edema in DR patients, making it the preferred option for treating diabetic macular edema. A meta-analysis has also shown that anti-VEGF pretreatment 6–14 days prior to vitrectomy in DR patients can reduce operation time, improve postoperative best-corrected visual acuity (BCVA), and decrease the recurrence rate of vitreous hemorrhage (51).

When anti-VEGF therapy is not successful, corticosteroids may be considered due to the involvement of multiple inflammatory mediators in the pathological processes in the retina (52). Common corticosteroids include dexamethasone, fluoroquinolone acetonide, and triamcinolone acetonide. Dexamethasone intravitreal implant not only improves patients’ symptoms but also reduces the psychological burden of frequent vitreous injections. However, it is important to be aware of the risk of increased intraocular pressure and cataract progression when using hormonal drugs (53). Persistent vitreous hemorrhage may require a vitrectomy to preserve functional vision (54). Blocking the expression of DR-related genes can help prevent neurovascular abnormalities in DR (36, 55, 56) while inducing stem cell differentiation can reshape retinal function (57). However, further study is needed before this technology can be applied in the clinic.

#### 4.3.4. Fundus image for deep learning

Diabetic retinopathy has different fundus manifestations in different stages. In the non-proliferative diabetic retinopathy (NPDR) stage, fundus manifestations include arteriolar hemangiomas, punctate retinal hemorrhage, hard exudation, cotton-wool spots, retinal edema, and beaded venous dilatation. Angiogenesis is the main feature of the fundus in the PDR stage. Fundus images can be used to detect and diagnose macular edema and vitreous hemorrhage, which are the main complications of DR. If the fundus image shows vitreous membranes with retinal eminence, it suggests that DR has developed tractional retinal detachment. Currently, fundus images of DR are not only used for clinical diagnosis but also for deep learning, to improve the detection rate and accuracy of DR.

### 4.4. The research frontier of DR

Keyword burst analysis provides a useful tool to forecast research frontiers and predict fundamental and clinical research trends. The current research frontiers of DR can be summarized as follows:

#### 4.4.1. Deep learning and AI models

The development and validation of deep learning algorithms for DR is the most commonly cited article (9). The improvements in algorithmic DR research have led to significant breakthroughs in the early diagnosis of DR. A new model has been developed to predict the development of diabetes into DR (58). Through deep learning of vast fundus images, AI has achieved early identification and severity grading of DR (59–63) and constantly optimized the algorithm to improve the detection rate and accuracy. In addition, AI can be used to evaluate prognosis and develop personalized treatment plans based on the personal electronic records of DR patients (64). The rapid development of technology has led to the incorporation of AI for early detection of DR into the American Diabetes Association’s guidelines (65). Combining AI and telemedicine can reduce public health costs and improve the efficiency of diagnosis and treatment, which will address the number imbalance between ophthalmic doctors and patients and be an important strategy to deal with the high global incidence of DR (66). However, an ethical review of a large number of original data is required for the research of AI algorithms, and the application of clinical care must be properly standardized and deployed (67, 68). Furthermore, there is an urgent need to establish a liability system for critical medical negligence, which may be caused by AI misdiagnosis and missed diagnosis (69).

#### 4.4.2. Biomarkers and OCTA

Biomarkers can be objectively measured and used as indicators to assess normal biologic status, pathogenic processes, or response to interventions. Abnormal results of the fundus, blood markers, cytokines, etc. can all be used as the biomarkers of DR. Identifying these biomarkers can assist doctors in diagnosis and timely intervention to avoid the aggravation of the patient’s condition. Furthermore, DR biomarkers, abnormal gene expression (70, 71), and metabolomics (72) may explain the pathophysiology, forecast the prognosis, and provide suggestions for novel therapeutic development.

In addition to biomarkers, OCTA, a non-invasive fundus imaging technique, can be utilized to classify DR objectively. OCTA is commonly used in conjunction with DR biomarker identification because it can provide a layered view of the retinal and choroidal vasculature in living tissue (73, 74). In OCTA pictures, biomarkers of DR were observed as the expansion of the macula without perfusion, a reduction in vessel density, and vascular structural alterations in the fundus (75–77). The development of wide-angle OCTA has increased the sensitivity of spotting non-perfusion regions and neovascular arteries in the retina (78). Moreover, associated fluorescein angiography (FA) images may be used to align the OCTA. Automatic positioning methods facilitate quantitatively comparing the microvascular characteristics in DR (79).

In conclusion, OCTA is extensively utilized in clinical and research applications. It may be used for clinical assessment, to track changes in fundus conditions, and to evaluate the efficacy of therapy. Therefore, developing DR screening programs and discovering more specific and sensitive biomarkers is vital to assist early identification of DR to minimize the incidence of visual impairment and blindness.

### 4.5. Strengths and limitations

This study utilized bibliometrics to evaluate publishing trends, leading research countries, institutes, and journals in the area of DR. The use of VOSviewer allowed for the identification of national collaborations and keywords with high frequency. CiteSpace illustrated changes in research hotspots and predicted future research trends. However, the limitation of this study is that it only included literature from a single database, which may have omitted relevant DR-related literature and introduced bias.

## 5. Conclusion

The results of this bibliometric analysis demonstrate that DR is a critical research field that has been expanding rapidly. The United States has had a significant academic impact on DR research, and international collaborations are increasingly important for the field’s development. OCTA screening and the identification of specific biomarkers are crucial for early DR detection and the prevention of visual impairment and blindness. With the increasing prevalence of DR, the development of AI technologies and telemedicine to address the shortage of ophthalmologists is a potential research hotspot and an urgent issue. These findings can provide valuable references for future DR research.

## Data availability statement

The raw data supporting the conclusions of this article will be made available by the authors, without undue reservation.

## Author contributions

HX and JT conducted the bibliometric analysis and drafted the manuscript. JD and FZ reviewed the manuscript. LL developed the search strategy. ML, MC, JZ, XW, and YN contributed to the data analysis. All authors contributed to the article and approved the submitted version.

## Conflict of interest

The authors declare that the research was conducted without any commercial or financial relationships that could potentially create a conflict of interest.

## Publisher’s note

All claims expressed in this article are solely those of the authors and do not necessarily represent those of their affiliated organizations, or those of the publisher, the editors and the reviewers. Any product that may be evaluated in this article, or claim that may be made by its manufacturer, is not guaranteed or endorsed by the publisher.
